# Prevalence of Osteoporosis and Its Associated Factors among Older Men with Type 2 Diabetes

**DOI:** 10.1155/2013/285729

**Published:** 2013-01-17

**Authors:** Hai-ling Chen, Li-li Deng, Ju-fen Li

**Affiliations:** Department of Endocrinology, Jishuitan Hospital, Beijing 100035, China

## Abstract

This study investigated the prevalence of osteoporosis and its associated factors in old men with T2DM to identify risk factors for low BMD. We enrolled 93 old men (≥60 years of age) with T2DM and 125 healthy old men (controls) and collected data of their lifestyle, medical history, bone densitometry, body weight, height, and blood pressure. Blood samples were collected for biochemical analyses. Urine samples were collected to determine 24 h urinary creatinine, albumin, and protein. Although no differences in age, blood pressure, waist-to-hip ratio, body mass index (BMI), and testosterone levels were observed, the prevalence of low BMD was significantly higher in the T2DM group compared to the control group. The risk of developing low BMD and fracture in T2DM subjects was increased by 46- and 26-fold, respectively, compared to control subjects. BMD of total spine and hip was positively correlated with BMI and negatively correlated with age, duration of diabetes, creatinine, and 24 h urinary albumin. So old men with T2DM have a greater risk of developing low BMD than old men without T2DM.

## 1. Introduction

Osteoporosis is characterized by low bone mass and microarchitectural deterioration of bone tissue, increasing susceptibility to fracture [1]. Although osteoporosis is often described as a silent disease because it is typically asymptomatic until a fracture occurs, the disease negatively and significantly impacts morbidity and mortality as it can lead to severe pain, deformity, disability, and death [1, 2]. While traditionally considered a condition afflicting predominantly postmenopausal women, osteoporosis is also an underrecognized disorder in men [3, 4] as analysis of hip fracture incidence indicates that one-third occur in men [5]. As compared to females, males often develop fractures 10 years later in life [4] and are less likely to survive with a mortality rate as high as 37.5% after hip fracture [6, 7].

Detection of osteoporosis in men is complicated by the fact that the average bone mineral density (BMD) in men who suffer a hip fracture is higher than that observed in women [8]. Compared to women, osteoporosis in men may often be due to secondary causes including corticosteroid therapy, excessive alcohol use, hypogonadism, low calcium intake, and cigarette smoking [3]. Hence, identifying factors related to increased risk of developing osteoporosis in men is critical to its prevention and management.

Like osteoporosis, type 2 diabetes mellitus (T2DM) is also highly prevalent in aging populations, causing substantial morbidity and mortality. Demographic trends with longer life expectancy and a lifestyle characterized by low physical activity and high-energy food intake contribute to the increasing incidence of diabetes mellitus and osteoporosis [9]. Some studies on the etiological association between diabetes and osteoporosis have reported that bone health is compromised by diabetes [10, 11]. Conversely, other studies of patients with T2DM revealed an increased fracture risk due to the risk of falling despite higher BMD [12]. Furthermore, meta-analyses indicate increased risk of both hip and vertebral fracture in T2DM patients with normal BMD [13]. Therefore, T2DM patients, particularly old patients, should be routinely screened for risk factors for developing osteoporosis as part of their disease management; however, factors other than BMD also need to be considered in assessing fracture risk in this population.

Because few studies have assessed the prevalence of osteoporosis in old male Chinese patients with T2DM [14, 15], the present study sought to investigate osteoporosis prevalence, assess levels of bone metabolism markers, and evaluate risk factors for low BMD. Because the effects of T2DM on bone metabolism are less clear in old men with T2DM, identifying causative factors and/or markers of osteoporosis beyond BMD analysis may contribute to its prevention and intervention in this population.

## 2. Materials and Methods

### 2.1. Study Participants

In this prevalence study, 381 men who were hospitalized in the Department of Endocrinology in the Jishuitan Hospital in Beijing, China, from March 2007 to February 2008 were screened for enrollment. These T2Dm patients were hospitalized to monitor their switch from oral antiglycemics to insulin therapy or when substantial adjustments in insulin dose were undertaken. The following inclusion criteria were applied for enrollment: (a) age ≥ 60 y and (b) diagnosis of T2DM according to the World Health Organization criteria [16]. Patients with the following characteristics were excluded from this study: body mass index (BMI) <19 kg/m^2^; heavy cigarette smoking (>20 cigarettes/day); heavy alcohol consumption (>5 beer equivalent drinks per day); heavy consumption of caffeinated beverages (>5 brewed tea equivalent drinks per day); use of drugs that interfere with bone and calcium metabolism, such as sex steroids, vitamin D metabolites, calcitonin, bisphosphonates, thyroid hormone, thiazolidinediones, heparin, warfarin, vitamin K, thiazides, and anticonvulsants; diseases that affect bone and calcium metabolism, such as thyrotoxicosis, rheumatoid arthritis, systemic lupus erythematosus, Cushing syndrome, hypogonadism, primary or secondary hyperparathyroidism, cirrhosis, malignancy, other nutrition and metabolism diseases and renal insufficiency; and those who were bedridden. In total, 288 of 381 patients were ruled out based on the above criteria and the other 93 were investigated.

Control subjects were selected from 216 healthy men (age ≥ 60 y), who were undergoing a routine annual medical checkup at the outpatient clinic of the Jishuitan Hospital during the same period. Of those screened, 125 subjects met the inclusion and exclusion criteria and were included as control subjects. Additionally, control subjects who had a past history or family history of diabetes mellitus were excluded, and blood glucose levels were confirmed using an oral glucose tolerance test (OGTT).

The study procedure was in accordance with the Helsinki Declaration and was approved by the Institutional Review Board of the Jishuitan Hospital. All participants provided written informed consent prior to enrollment.

### 2.2. Clinical Assessment

Study participants completed a comprehensive 130-question survey detailing their medical history, particularly relating to osteoporosis (e.g., past fractures, symptoms of osteoporosis, and degree of exposure to sun), family medical history, and lifestyle, including cigarette smoking, alcohol use, tea consumption, level of physical activity, diet, dietary calcium consumption, oral calcium supplementation, comorbidities, and daily pharmacotherapy. For the patients with T2DM, duration of diabetes, presence of diabetic microvascular and macrovascular complications, antidiabetic therapy, and degree of blood glucose control prior to hospitalization were also determined.

Lifestyle parameters were defined as follows: physically active was defined as engaging in strenuous exercise for more than 3 days per week to achieve maximum heart rate; exposure to >0.5 h sunlight per day was considered as sufficient sunlight exposure; a smoker was defined as individual currently smoking at least 1 cigarette/day or >18 packs/y, and those who had ceased smoking for >1 y were considered a nonsmoker; as alcohol user was defined as individual consuming >2 drinks/week (beer equivalent) for more than a year; staple food consumption was defined as the amount (g) of starchy foods (e.g., rice, noodles, bread, etc.) consumed daily.

Body weight, height, and waist and hip circumference were measured. Body mass index (BMI) and waist-to-hip ratio were calculated. Blood pressure was measured after 10 min rest in a supine position with a sphygmomanometer. Blood pressure values were the means of three readings taken at each of three examinations.

### 2.3. Bone Densitometry and Laboratory Tests

BMD was measured at the lumbar spine and femur in all study participants using Lunar Prodigy (GE Corporation, Fairfield, CT, USA) dual-energy X-ray absorptiometry (DEXA) scanner and analyzed according to WHO criteria [17]. T scores were calculated using normal BMD ranges obtained from a healthy Chinese population. As defined by the WHO, osteopenia is defined as a bone density between 1 and 2.5 standard deviations below the mean value while and osteoporosis was considered a bone density 2.5 standard deviation below the mean value for a young adult at the lumbar spine (L1–L4), total hip, and femoral neck based on T scores. The BMD results were expressed in g/cm^2^.

Fasting blood samples were drawn from the median cubital vein in the morning. Fasting blood glucose (FBG), postprandial blood glucose (2 h-PBG), hemoglobin A1c (HbA_1_C), serum calcium (Ca^2+^), serum phosphorus (P), alkaline phosphatase (ALP), triglyceride (TG), total cholesterol (TC), low-density lipoprotein cholesterol (LDL), high-density lipoprotein cholesterol (HDL), uric acid (UA), serum creatinine (SCr), blood urea nitrogen (BUN), alanine transaminase (ALT), and aspartate transaminase (AST) levels were measured on a Hitachi 7600 Automatic biochemical analysis instrument (Hitachi, Tokyo, Japan). Fasting insulin levels were determined using an electrochemiluminescence method following manufacturer's instructions (Abbott, Abbott Park, IL). In addition, 24 h urine samples were collected, and urinary creatinine, albumin, and protein were measured on the Hitachi 7600 Automatic biochemical analysis instrument. Creatinine clearance rate (ClCr) was calculated using the Cockcroft-Gault formula.

The serum concentration of 25-hydroxyvitamin D, calcitonin, insulin-like growth factor-1, osteocalcin, bone-specific alkaline phosphatase, tartrate-resistant acid phosphatase-5b, and urinary hydroxyproline were measured using enzyme-linked immunosorbent assays (ELISA) following the manufacturer's instructions (Immunodiagnostic Systems, Boldon, UK). Serum testosterone and parathyroid hormone levels were determined using chemical bioluminescent assays following manufacturer's instructions (Beckman Coulter, USA).

### 2.4. Statistical Analysis

Continuous data were expressed by mean and standard deviation (SD); categorical data were expressed by count and percentages. The comparisons for continuous data between groups were performed using the independent two-sample *t*-test. The differences in frequencies of categorical data between groups were tested by the Chi-square test. The correlations between T2DM-related measurements and BMD were performed using the Pearson correlation coefficient. The statistical significance was set at a *P* value ≤ 0.05. The SPSS 15.0 statistical software (SPSS Inc., Chicago, IL, USA) was used for all statistical analyses.

## 3. Results

### 3.1. General Patient and Lifestyle Characteristics

The mean duration of diabetes in the T2DM group was 10.5 ± 6.1 y. As shown in Table 1, the general characteristics of the T2DM and control groups were compared. No difference in age was observed between the T2DM group (mean age 68.3 y, range 60–79 y) and the control group (mean age 69.4 y, range 60–80 y). In addition, patient height, weight, BMI, waist/hip ratio, waist circumference, hip circumference, and blood pressure were comparable between the two groups (*P* > 0.05). Significantly higher FPG, PBG, and HbA_1_C levels were detected in the T2DM group as compared to the control group (Table 1; *P* < 0.05).

In addition to general characteristics, lifestyle factors were compared between the two groups (Table 1). There were significantly less individuals defined as current smokers (25.8% versus 45.6%, *P* = 0.003) and alcohol drinkers (28.0% versus 42.4%, *P* = 0.033) in the T2DM group than in the control groups, respectively. In addition, study participants in the T2DM group had a significantly shorter duration of cigarette smoking than the control subjects (*P* = 0.045). Furthermore, compared to the control subjects, the T2DM group consumed significantly less staple foods (*P* < 0.001). Finally, the T2DM group was significantly more physically active than the control group (*P* < 0.001).

### 3.2. Prevalence of Osteoporosis

BMD was assessed and compared in both groups (Table 2). The prevalence of low BMD was significantly higher in the T2DM group as compared to the control group (59.1% versus 24.0%, respectively; *P* < 0.0001). Low BMD was significantly associated with T2DM with an odds ratio of 4.6 (95% confidence interval: 2.6–8.2).

### 3.3. Effects of T2DM on Bone Metabolism

BMD and T score measurements at the lumbar spine and femur of the two groups were summarized in Table 3. Significantly lower T score of femoral neck (*P* = 0.006), trochanter major T score and BMD (T Score: *P* = 0.023 and BMD: *P* = 0.021), Ward's triangle T score and BMD (T score: *P* = 0.023 and BMD: *P* = 0.036), and femur total BMD (*P* = 0.012) were observed in the T2DM group as compared to the control group. Total spine (L1–L4) BMD was also significantly lower in the T2DM patients than in the control subjects (0.7 versus 1.1 g/cm^2^, *P* = 0.015).

To determine if the altered BMD scores correlated with markers of bone resorption and formation, the concentration of those markers were determined and compared in both groups (Table 3). Most markers of bone resorption and bone formation were significantly lower in the T2DM group as compared to the control group (*P* < 0.05). However, bone-specific alkaline phosphatase and urinary hydroxyproline/creatinine levels were similar between the two groups. As for other markers that may alter bone metabolism, testosterone and 25-hydroxy vitamin D_3_ levels were similar between the two groups. Significantly higher calcitonin (*P* = 0.034), parathyroid hormone (*P* = 0.042), and serum phosphorus (*P* = 0.031) were detected in T2DM patients as compared to control subjects. Alternatively, insulin-like growth factor I and serum calcium levels were significantly lower in the T2DM group as compared to the control subjects (*P* = 0.042 and *P* = 0.048, resp.; Table 3).

Duration of lumbago and back pain in the T2DM group was significantly longer than that of the control subjects (*P* < 0.001). In addition, the frequency of fracture was significantly higher in the T2DM group (*P* = 0.004). However, no differences in the duration of arthralgia and the age of first fracture were observed (Table 3).

### 3.4. Effects of T2DM on Biochemical Markers

As shown in Table 4, a comparison of common laboratory indices was undertaken. Although significant differences in ALT, AST, BUN, creatinine, UA, TC, TG, LDL, and ClCr were observed between the two groups, all remained within normal ranges (*P* < 0.05). In addition, both the 24 h urinary albumin and protein were significantly higher in the T2DM group (*P* = 0.005 and *P* = 0.006, resp.). Compared with the control subjects, the T2DM group had a significantly lower ClCr (*P* = 0.042) though it was also within the normal range. The serum albumin/globulin and HDL levels were similar between the two groups (Table 4).

### 3.5. Correlations between Type 2 Diabetes Related Factors and BMD

Correlation analysis revealed that the BMD of total spine (L1–L4) was positively correlated with duration of physical activity and BMI and was negatively correlated with age, duration of diabetes, creatinine, and 24 h urinary albumin (all *P* ≤ 0.029; Table 5). No correlation was observed between BMD of total spine (L1–L4) and waist-to-hip ratio, serum calcium, FPG, TC, and LDL-C concentrations.

Whereas BMD of the total hip was positively correlated with BMI, waist-to-hip ratio, and serum calcium, it was negatively correlated with duration of diabetes, 24 h urinary albumin, FPG, TC, and LDL-C (*P* ≤ 0.031; Table 5). No significant correlation was found between BMD of both total hip and total spine (L1–L4) and cigarette smoking, tea or alcohol consumption, daily dietary staple food intake, HbA_1_C, blood pressure, ALT, AST, serum albumin, serum globulin, A/G, uric acid, AKP, serum phosphorus, TG, HDL-C, and testosterone (data not shown).

## 4. Discussion

In the present study, the prevalence of osteoporosis, bone metabolism marker levels, and risk factors for developing low BMD were analyzed in healthy old male patients and those with T2DM for a relatively long diabetic duration (10.5 ± 6.1 y). Significant differences in the prevalence of low BMD and T score measurements at the lumbar spine and femur, risk of developing low BMD and fracture, bone formation and resorption markers, and other markers known to affect bone metabolism were observed between the two groups. Finally, BMD of total spine (L1–L4) and hip was positively correlated with BMI and negatively correlated with age, duration of diabetes, and 24 h urinary albumin.

Patients with type 1 diabetes (T1DM) often have lower BMD as compared with healthy subjects, likely as a result of insulin deficiency [10]. However, conflicting studies regarding the association between osteoporosis and T2DM have been reported. Whereas some studies reported a higher prevalence of osteoporosis among individuals with T2DM [15, 18] even despite BMD [19], others have observed no differences in BMD between T1DM, T2DM, and control subjects [19]. Alternatively, another study reported that T2DM was a protective factor for osteoporosis [20]. In the present study, the prevalence of low BMD was 59.4% and 24.0% in the T2DM and control groups, respectively, which was similar to that reported by Xia et al. [15]. In addition, most T score and BMD measurements at the lumbar spine and femur in the T2DM group were significantly lower than observed in the control groups, even after adjusting for systolic blood pressure, cigarette smoking, and intake of daily dietary staple foods. This coincided with an increased incidence of osteopenia or osteoporosis in the T2DM group. Moreover, the risk of developing low bone mass for the T2DM patients analyzed was 4.583-fold higher than in the control group. These results suggest that T2DM itself may be a risk factor for low bone mass and are similar to those of Sta and Li-Yu [21], who reported a prevalence of T2DM in subjects with osteoporosis of 22.4% compared to 19.1% in those with normal BMD. The conflicting results in patients with T2DM are likely due to the pathogenetic complexity of the condition [9], including differences in the duration, severity, and treatment as well as different methods used to measure BMD [22]. In addition, the investigated populations varied in age, BMI, gender, insulin levels, and duration of disease, which might also result in the contradictory results of previous studies [23].

Although the relationship between osteoporosis and T2DM remains to be fully elucidated, patients with T1DM or T2DM are at greater risk of fractures due to decreased BMD [24]. Interestingly, a high BMI is associated with increased BMD and has been shown to be a protective factor against the risk of osteoporosis and fracture in some studies [25–27]. However, Xu et al. [14] reported a positive correlation between weight as well as BMI with BMD in Chinese male patients with T2DM. The results of this study are in agreement with results of a meta-analysis that found that BMI as a predictor of BMD also holds true in patients with T2DM [28]. In the present study, the risk of fracture for the T2DM group was 2.561-fold higher than that of the control subjects. Lower BMD and impaired bone formation may be caused by insulinopenia and hyperglycemia associated with T2DM [22], which is consistent with the high levels of FPB, PBG, and HbA_1_C and reduced T score and BMD observed in the T2DM group.

In the present study, the T score measured at the femoral neck showed the greatest difference between the two groups, which is similar to the Health ABC Study that reported an association between T2DM and rapid bone loss at the femoral neck [29]. The femoral neck may be more vulnerable to the metabolic alterations characteristic of T2DM, as cortical and cancellous bone may be differently affected by insulin, glucose, BMI, sex steroids, and parathyroid hormone [18].

The present study assessed the BMD of both the hip and spine (L1–L4). Although the reference site is traditionally the hip, combining the BMD results at more than one region from a single scan significantly increases fracture risk prediction [30]. Moreover, osteoporotic vertebral fractures may be accompanied with osteopenia or even normal peripheral BMD in some cases, possibly because trabecular bone in the spine has a higher turn-over rate than cortical bone in the periphery [31]. 

Although osteoporosis is asymptomatic until fracture, fractures are often undetectable and misdiagnosed, and few studies have assessed the related symptoms of osteoporosis. The duration of arthralgia and age of first fracture were similar between groups in the present study; however, the T2DM group had a significantly longer duration of lumbago and back pain, which might be due to the high incidence of osteopenia and osteoporosis. Because patients with diabetes are less sensitive to pain due to diabetic neuropathy, the pain associated with osteoporosis and the duration of arthralgia maybe more significant in this population. In Rasul et al. work [32], old male patients with diabetes and polyneuropathy experienced higher bone turnover than those without polyneuropathy, suggesting that prevention or treatment of polyneuropathy might reduce osteoporosis and fracture in T2DM. 

Although BMD as measured by DEXA is the best predictive and prognostic measure of osteoporosis, a comprehensive risk assessment for osteoporosis beyond BMD measurements should be undertaken in patients with diabetes [33]. Analysis of bone metabolism markers along with BMD is considered to improve fracture prediction, particularly those associated with bone turnover [34, 35], which has the added advantage of independently revealing fracture risk [30, 36]. Diabetic osteopenia is characterized by low bone turnover, resulting from osteoblast deficiency and subsequent reduced bone formation accompanied by normal bone resorption [22, 37]. In addition, the systemic changes associated with diabetes, including inflammation, advanced glycation end-product accumulation, and reactive oxygen species generation, may also affect bone remodeling [19]. In this study, both the markers of bone formation and resorption were significantly lower in the T2DM, which is indicative of low-bone-turn-over osteoporosis. These results also suggest that T2DM can affect bone resorption.

 The proposed reasons for diabetes-associated bone loss include calcium, phosphorus, magnesium, and trace element deficiency originating from poor blood glucose control, hypoinsulinemia, and chronic complication of diabetes [38, 39]. Since, in this study, the patients with T2DM had generally well-controlled disease, the influence of poorly-controlled T2DM on bone health could not be determined. However, previous studies have reported conflicting results regarding the influence of glycemic control on BMD [40, 41]. In addition, a recent study suggested that T2DM and osteoporosis were etiologically related through the actions of osteocalcin and adiponectin [42]. In the present study, significantly increased PTH and calcitonin and decreased serum calcium levels were observed in the T2DM group, which was similar to those in Inzerillo and Epstein [39]. However, the clinical significance of these differences remains to be determined as all were within the normal range.

Osteoporosis in men is often due to secondary causes in addition to advancing age, including hypogonadism, smoking, chronic obstructive pulmonary disease, glucocorticoid therapy, and androgen deprivation therapy for prostate cancer, and alcohol abuse [3, 4]. Meier et al. [43] reported that testosterone levels could also predict risk of fracture. In the present study, the testosterone levels of both groups were within the normal range. Because bone loss accelerates after the age of 70 years, and rapid bone loss is more common with deficient testosterone levels [44], differences in the age of the study population may account for the differential effects of testosterone levels. In addition to testosterone, serum levels of 25-hydroxyvitamin D_3_ below 25 ng/mL (62.5 mmol/L) were associated with an increased risk of hip fracture in men and women older than 65 years [45]. However, no differences in 25-hydroxyvitamin D_3_ levels were observed between the groups in our study, which is similar to a previous report [42], and suggests that altered 25-hydroxyvitamin D_3_ levels are not a major reason for bone loss.

IGF-1 can also stimulate osteoblast growth and the formation of bone matrix [46]. In men, trabecular bone loss is associated with changes in IGF-1 signaling [47]. Furthermore, high blood glucose level can inhibit the synthesis and release of IGF-1 [48]. IGF-1 levels were significantly lower in the T2DM group analyzed in the present study. However, its predictive value has yet to be determined. 

Many lifestyle factors can influence bone metabolism, and the negative effects of smoking, lower calcium consumption, and physical inactivity on osteoporosis have been reported [49, 50]. However, Anaforoglu et al. [23] reported no such effects. In the present study, there were fewer smokers and alcohol drinkers in the T2DM group; this group also consumed less daily dietary staple food and more individuals were physically active although no difference in duration of being physically active was observed.

BMD of hip and total spine (L1–L4) was positively correlated with duration of physically active and BMI, indicating a protective effect of BMI, which is similar to a previous study [20]. BMD of total hip was also positively correlated with waist-to-hip ratio and negatively associated with TC and LDL-C. The reasons for these associations remain unclear and require further analysis.

The present study has limitations. The sample size was relatively small, particularly in the T2DM group due to the strict inclusion and exclusion criteria. Further large-scale, prospective studies to assess diabetes-associated changes in BMD and its determinants are needed.

In conclusion, in old male patients with T2DM, altered bone formation and resorption may result in their increased risk of low BMD and subsequent low-bone-turn-over osteoporosis. Assessing of the markers of bone metabolism along with BMD and patient and lifestyle characteristics may help determine bone status in this population and contribute to the prevention and intervention of osteoporosis.

## Figures and Tables

**Table 1:** General characteristics of patients with T2DM and the control subjects.

	Control group (*n* = 125)	T2DM group (*n* = 93)	*P* value
General characteristics			
Age (y)	69.43 ± 5.20	68.26 ± 4.01	0.061
BMI (kg/m^2^)	24.29 ± 4.48	26.42 ± 3.90	0.069
Height (cm)	166.91 ± 5.27	167.31 ± 6.31	0.611
Weight (kg)	67.74 ± 14.21	74.12 ± 12.37	0.052
Waist-to-hip ratio	0.93 ± 0.24	0.94 ± 0.25	0.055
Waist circumference (cm)	93.55 ± 10.05	94.77 ± 10.53	0.633
Hip circumference (cm)	100.71 ± 9.68	102.93 ± 7.79	0.126
Systolic pressure (mmHg)	120.00 ± 11.06	135.11 ± 13.55	0.051
Diastolic pressure (mmHg)	80.00 ± 12.09	85.11 ± 11.15	0.053
FPG (mmol/L)	4.88 ± 1.83	7.76 ± 2.52	0.004**
PBG (mmol/L)	7.92 ± 2.58	11.91 ± 2.85	0.016*
HbA1C (%)	4.96 ± 1.02	7.18 ± 1.06	0.004**
Lifestyle factors			
Current smokers	57 (45.6%)	24 (25.8%)	0.003**
Duration of cigarette smoking (y)	20.08 ± 18.96	14.73 ± 13.92	0.045*
Alcohol users	53 (42.4%)	26 (28.0%)	0.033*
Duration of alcohol use (y)	9.69 ± 8.40	7.58 ± 7.50	0.101
Duration of tea consumption (y)	29.49 ± 15.69	35.74 ± 17.55	0.062
Tea consumption (g/y)	1014.0 ± 784.5	1024.0 ± 1022.0	0.955
Staple food consumption (g/day)	352.5 ± 101.0	288.5 ± 71.5	<0.001**
Calcium intake of >1000 mg/day	26 (20.8%)	19 (20.4%)	0.543
Physically active^#^	46 (36.8%)	85 (91.4%)	<0.001**
Duration of physical activity (y)	3.72 ± 1.70	3.76 ± 1.22	0.775
Sufficient exposure to sun^&^	123 (98.4%)	91 (97.8%)	0.765

T2DM: Type 2 diabetes mellitus; BMI: body mass index; FPG: fasting plasma glucose; PBG: postprandial blood glucose.

*P < 0.05; **P < 0.01.

Data are expressed as mean ± SD.

^#^Subjects engaging in strenuous exercise for more than 3 days per week were reported as being physically active.

^&^Subjects exposed to sunlight for more than 0.5 h/day were reported as having sufficient exposure.

**Table 2:** Prevalence of osteoporosis in patients with T2DM and control subjects.

	Control group (*n* = 125)	T2DM group (*n* = 93)	*P* value	OR (95% CI)
BMD				
Normal BMD	95 (76.0%)	38 (40.86%)	<0.001**	Reference
Low BMD	30 (24.0%)	55 (59.14%)		4.583 (2.560–8.207)
Osteopenia	28 (22.4%)	24 (25.81%)		
Osteoporosis	2 (1.60%)	31 (33.33%)		

T2DM: type 2 diabetes mellitus; BMD: bone mineral density; OR: odds ratio; CI: confidence interval.

**P < 0.01.

**Table 3:** Effects of T2DM on bone mineral density and bone metabolism.

	Control group (*n* = 125)	T2DM group (*n* = 93)	*P* value
Hip			
Femoral neck T score	0.03 ± 1.37	−0.63 ± 2.03	0.006^∗∗#^
Femoral neck BMD (g/cm^2^)	0.83 ± 0.08	0.81 ± 0.13	0.382^#^
Trochanter major T score	0.33 ± 1.36	−0.22 ± 1.98	0.023^∗#^
Trochanter major BMD (g/cm^2^)	0.71 ± 0.07	0.65 ± 0.13	0.021^∗#^
Ward's triangle T score	−0.90 ± 1.32	−1.41 ± 1.89	0.023^∗#^
Ward's triangle BMD (g/cm^2^)	0.55 ± 0.08	0.42 ± 0.15	0.036^∗#^
Femur total T score	0.49 ± 1.26	0.34 ± 1.65	0.438^#^
Femur total BMD (g/cm^2^)	0.82 ± 0.29	0.68 ± 0.24	0.012^∗#^
Lumbar spine			
Total spine (L1–L4) T score	0.83 ± 1.43	0.45 ± 2.05	0.137^#^
Total spine (L1–L4) BMD (g/cm^2^)	1.08 ± 0.26	0.72 ± 0.21	0.015^∗#^
Markers of bone resorption			
Tartrate-resistant acid phosphatase-5b (U/L)	4.65 ± 1.33	3.28 ± 1.47	0.027*
Urinary hydroxyproline/creatinine (g/mol)	50.30 ± 19.63	45.09 ± 23.59	0.160
Markers of bone formation			
Osteocalcin/bone gla protein (ng/mL)	9.10 ± 3.97	5.09 ± 3.41	0.031*
Bone-specific alkaline phosphatase (*μ*g/L)	9.14 ± 5.62	9.31 ± 4.42	0.798
Alkaline phosphatase (IU/L)	72.40 ± 9.70	59.00 ± 16.53	0.017*
Other markers that may affect bone metabolism			
Calcitonin (pg/mL)	2.80 ± 0.97	8.73 ± 7.45	0.034*
Parathyroid hormone (pmol/L)	3.44 ± 1.76	4.60 ± 2.05	0.042*
25-hydroxy vitamin D_3_ (nmol/L)	39.30 ± 16.32	47.78 ± 18.17	0.052
Insulin-like growth factor I (ng/mL)	169.90 ± 29.8	121.88 ± 32.18	0.042*
Serum calcium (mmol/L)	2.46 ± 0.56	2.25 ± 0.49	0.048*
Serum phosphorus (mmol/L)	1.11 ± 0.36	1.22 ± 0.21	0.031*
Testosterone (nmol/L)	9.98 ± 5.08	11.41 ± 4.77	0.073
Symptoms of osteoporosis			
Duration of lumbago and back pain (y)	2.97 ± 1.68	11.11 ± 5.27	<0.001**
Duration of arthralgia (y)	5.91 ± 1.61	6.67 ± 3.26	0.689
Fracture	18 (14.4%)	28 (30.1%)	0.004**
Age of first fracture (y)	47.64 ± 6.31	47.17 ± 22.59	0.916

T2DM: type 2 diabetes mellitus; BMD: bone mineral density.

*P < 0.05; **P < 0.01.

Data are expressed as mean ± SD.

^#^Adjusted for systolic pressure, cigarette smoking, and daily intake of dietary staple foods.

**Table 4:** Laboratory test results of patients with T2DM and the control subjects.

	Control group (*n* = 125)	T2DM group (*n* = 93)	*P* value
ALT (IU/L)	33.40 ± 9.70	15.11 ± 6.00	<0.001**
AST (IU/L)	29.40 ± 7.59	14.82 ± 3.64	<0.001**
Serum albumin (g/L)	42.82 ± 6.38	38.08 ± 8.34	0.079
Serum albumin/globin	1.68 ± 0.15	1.59 ± 0.21	0.131
BUN (mmol/L)	4.70 ± 0.84	6.07 ± 2.37	0.003**
Creatinine (*μ*mol/L)	45.40 ± 11.38	73.27 ± 26.11	0.002**
UA (*μ*mol/L)	271.80 ± 17.29	303.71 ± 57.46	0.003**
TC (mmol/L)	3.24 ± 0.98	4.26 ± 0.91	0.034*
TG (mmol/L)	0.96 ± 0.49	1.52 ± 0.84	0.041*
HDL-C (mmol/L)	0.99 ± 0.42	1.16 ± 0.41	0.051
LDL-C (mmol/L)	1.74 ± 0.55	2.19 ± 0.45	0.033*
24-h urinary albumin (mg)	13.34 ± 6.01	473.69 ± 261.14^a^	0.005**
24-h urinary protein (mg)	101.20 ± 49.08	730.11 ± 233.43^b^	0.006**
ClCr (mL/min)	104.81 ± 29.26	84.85 ± 35.38	0.042*

T2DM: type 2 diabetes mellitus; ALT: alanine aminotransferase; AST: aspartate aminotransferase; BUN: blood urea nitrogen; UA: uric acid; TC: total cholesterol; TG: triglyceride; HDL-C: high-density lipoprotein cholesterol; LDL-C: low-density lipoprotein cholesterol; ClCr: creatinine clearance rate.

*P < 0.05; **P < 0.01.

Data are expressed as mean ± SD.

^a^higher than 30 mg/24 h; ^b^higher than the normal range of 0–150 mg.

**Table 5:** Correlations betwteen T2DM-related factors and BMD.

	Pearson correlation coefficient (*P* value)	
	BMD of total spine (L1–L4)	BMD of total hip
BMI	0.448 (*P* < 0.001)**	0.342 (*P* = 0.019)*
Duration of physical activity (y)	0.250 (*P* < 0.001)**	—
Waist-to-hip ratio	—	0.316 (*P* < 0.001)**
Serum calcium	—	0.293 (*P* = 0.031)*
Age (y)	−0.151 (*P* = 0.029)*	−0.002 (*P* = 0.018)*
Duration of diabetes (y)	−0.314 (*P* < 0.001)**	−0.421 (*P* < 0.001)**
24 h urinary albumin (mg)	−0.640 (*P* < 0.001)**	−0.80 (*P* < 0.001)**
Creatinine (*μ*mol/L)	−0.401 (*P* = 0.002)**	—
FPG (mmol/L)	—	−0.463 (*P* < 0.001)**
TC (mmol/L)	—	−0.393 (*P* = 0.003)**
LDL-C (mmol/L)	—	−0.345 (*P* = 0.010)*

T2DM: type 2 diabetes mellitus; BMD: bone mineral density; BMI: body mass index; FPG: fasting plasma glucose; TC: total cholesterol; LDL-C: low-density lipoprotein cholesterol.

*P < 0.05; **P < 0.01.
